# Preference, Attitude, Recognition and Knowledge of Fruits and Vegetables Intake Among Malay Children

**DOI:** 10.21315/mjms2020.27.2.11

**Published:** 2020-04-30

**Authors:** Mashitah Sha An Ali, Nur Azreen Mohd Nazir, Zahara Abdul Manaf

**Affiliations:** Dietetics Programme, Faculty of Health Sciences, Universiti Kebangsaan Malaysia, Kuala Lumpur, Malaysia

**Keywords:** preference, attitude, recognition and knowledge, cross-sectional study, fruit intake, vegetable intake, children

## Abstract

**Background:**

The low consumption of fruits and vegetables among children is a global challenge. Foods recognition, nutrition knowledge and attitude are factors that influence children’s dietary practices. This study aims to assess the preference, attitude, recognition and knowledge of fruits and vegetables intake among Malay children.

**Methods:**

A cross-sectional study was conducted among Malay children from five primary schools in Kuala Lumpur using self-administered questionnaires.

**Results:**

A total of 134 Malay children (70 males and 64 females) with a mean (SD) age of 10.3 (1.0) years were recruited. Majority of the children had a father (61.9%) and a mother (56.0%) with secondary school education and earned below RM3,900 (70.9%) per month. The most preferred fruits and vegetable were bananas (91.9%) and carrots (71.4%), while the most recognised was oranges (100.0%) and tomatoes (96.3%). The children demonstrated an overall moderate level of attitude, recognition and knowledge with mean (SD) scores of 70.3 (19.9), 76.8 (18.1) and 73.6 (17.5), respectively, towards fruits and vegetables intake. Majority of the children (53.0%) were not aware of the daily recommended servings of fruits and vegetables, while 40.0% of children expressed a low attitude towards eating a variety of fruits and vegetables. The willingness to try a new type of vegetables and consume more vegetables was lower (68.7%) compared to fruits (75.4%).

**Conclusion:**

The preferences and recognition of fruits were higher compared to vegetables among the children. The children demonstrated a moderate level of attitude, recognition and knowledge towards fruits and vegetables consumption. Efforts to educate children on the recommended number of servings per day and improve their acceptability of vegetables should be implemented to promote the increase in fruits and vegetables consumption among children.

## Introduction

The consumption of fruits and vegetables plays an important role in providing a diversified and nutritious diet due to its highly beneficial nutrient content such as vitamins, minerals, phytochemicals and fibres (1–2). Several studies have shown that the increased consumption of fruits and vegetables reduces the risks of obesity, cardiovascular diseases, cancers and all-cause mortality (3–5). Besides, regular consumption of fruits and vegetables may prevent overweight and obesity among children and adolescents (6) due to its low energy density and glycaemic index properties (7) that maintain healthy body weight.

The Malaysian Dietary Guidelines for Children and Adolescents recommends that Malaysian children and adolescents aged between 7 and 18 years should eat at least three servings of vegetables and two servings of fruits daily (8). Nevertheless, despite the significant health benefits of fruits and vegetables, the consumption of fruits and vegetables among children worldwide remains low (9–11). In Malaysia, the average daily intake of fruits and vegetables among children below 7 years old is only 0.91 and 1.07 servings of fruits and vegetables, respectively (12), which is lower than the recommendation of five servings per day. Malay children showed the lowest mean daily intake of vegetables (0.87 servings) compared to Chinese (1.47 servings) and Indian (1.86 servings) children.

It is crucial to establish a healthy eating pattern during childhood since these eating habits are likely to be maintained throughout their adolescence and later on into adulthood (13–14). In most cases, children tend to eat a particular food that is familiar to them (15). This is directly related to the family environment, daily exposure to fruit and vegetables and their parents’ dietary patterns (16–18). Sensory aspects such as taste, colour and texture were also found to influence the preference and consumption of fruits and vegetables among children (17, 19). The rejection of green leafy vegetables among children due to its bitter taste is thought to be an adaptation strategy by children as they experience new types of food (19). Therefore, recognition and preference are important determinants that influence the consumption of fruits and vegetables among children.

Increased knowledge and positive attitudes also have the potential to mediate behavioural changes based on the principles of social cognition theory (20). Nutrition intervention programmes such as school gardening activities are one of the strategies to improve children’s fruits and vegetables consumption as it increases a student’s knowledge, preferences and exposure towards fruits and vegetables (21–23). Therefore, the assessment of nutrition knowledge and attitudes among children towards fruits and vegetables intake is an important factor in tailoring an effective nutrition intervention programme. It was reported that a majority of Malaysian adolescents were not aware of the recommended daily intake of dietary fibre and the serving sizes of fruits and vegetables (24). However, little is known of the knowledge and attitudes regarding fruits and vegetables intake among Malaysian children.

To date, only one study has been performed in Malaysia to assess the knowledge, attitudes and practices regarding dietary fibre intake among adolescents (24). Since the dietary pattern evolves greatly during childhood, the understanding of factors that influence their dietary intake is crucial for the establishment of healthy eating habits among children. Therefore, this study aims to assess the preferences, attitudes, recognition and knowledge of fruits and vegetables intake among Malay children. It is envisaged that the findings of this study can be used to create an effective and relevant intervention programme for children to increase their fruits and vegetables intake.

## Methods

### Study Population and Design

A cross-sectional study was conducted among 134 Malay primary school students aged between 9 and 12 years that were conveniently sampled from five randomly selected primary schools in Kuala Lumpur. The sample size was calculated using a single proportion formula by Daniel (25) with the precision level and confidence interval set at 7% and 95%. The 7% level of precision was used due to the limited time and resources available while conducting this study (26). Additionally, the estimation of prevalence was set at 80% based on previous estimation of prevalence for Malaysian children who had an inadequate intake of fruits and vegetables (12). Therefore, a sample size of 139 was required for this study with an attrition rate of 10%.

Written approvals were obtained from the Malaysian Ministry of Education, State Education Departments and school administrators. An information sheet regarding the study was given to all subjects and informed consents were obtained from their parents or guardian. Children who were able to read and write as well physically and mentally fit were eligible for this study. The school teachers facilitated the selection of students since they were aware of the students who met all the inclusion criteria based on the mental, physical and cognitive developmental aspects required.

### Questionnaires

The children’s preferences, attitudes, recognition and knowledge of the fruits and vegetables intake were assessed using validated questionnaires adapted from previous studies (21–22, 24). The questionnaire was translated from English into the Malay language using the back-to-back translation method. An expert committee consisting of dietitians, nutritionists and schoolteachers were asked to review the translated questionnaire to ensure that it was well understood by the children. In addition, the questionnaire was pilot tested among 30 children aged between 9 and 12 years and minor amendments were made to the questionnaire as required. The children answered the questionnaire individually with researchers’ supervision.

The questionnaire regarding the preferences and recognition of fruits and vegetables was adapted from a previous study (21) and translated into the Malay language. Cronbach’s alpha coefficient for this questionnaire was 0.926. In this questionnaire, images of 26 types of readily available local Malaysian fruits and vegetables were included, consisting of 11 and 15 types of fruits and vegetables. The subjects were asked to write the name of the fruits and vegetables based on the images provided. One point was awarded for the correct answer, while the wrong answer resulted in a zero point. Furthermore, the subjects’ preference for each fruits and vegetables was assessed using ‘1= yes’ and ‘0 = no’ options.

Next, the questionnaire on attitude and knowledge regarding the fruits and vegetables intake was adapted from previous studies (22, 24) and translated into the Malay language. This questionnaire was validated and the Cronbach’s alpha coefficient values of 0.811 and 0.683 were obtained for the attitude and knowledge sections, respectively. The knowledge and attitude sections contained 11 questions each consisting of ‘1= yes’ and ‘0 = no’ options. For the knowledge section, images corresponding to the portion size of one serving of fruits and vegetables were included for questions related to ‘Everyone has to eat three servings of vegetables every day’ and ‘Everyone has to eat two servings of fruits every day’. The total scores for attitude, recognition, and knowledge were presented as percentages and graded into ‘low’, ‘moderate’ and ‘high’ categories. Children who scored < 40% for the attitude, recognition and knowledge sections were placed in the low category, while scores between 40% and 80% and higher than 80% were categorised as medium and high categories (24).

### Statistical Analysis

The statistical analysis of the data was performed using IBM SPSS Statistics Version 25. Descriptive analyses were performed to determine the percentages, means and standard deviations for the quantitative data collected in this study.

## Results

### Socio-Demographic Profile

A total of 134 Malay children participated in this study, comprising 52.2% males and 47.8% females with a mean (SD) age of 10.3 (1.0) years. Majority of the children had a father (61.9%) and a mother (56.0%) who obtained secondary school education and earned less than RM3,900 per month (70.9%) as shown in Table 1.

### Preferences for Fruits and Vegetables

**Figure 1:**
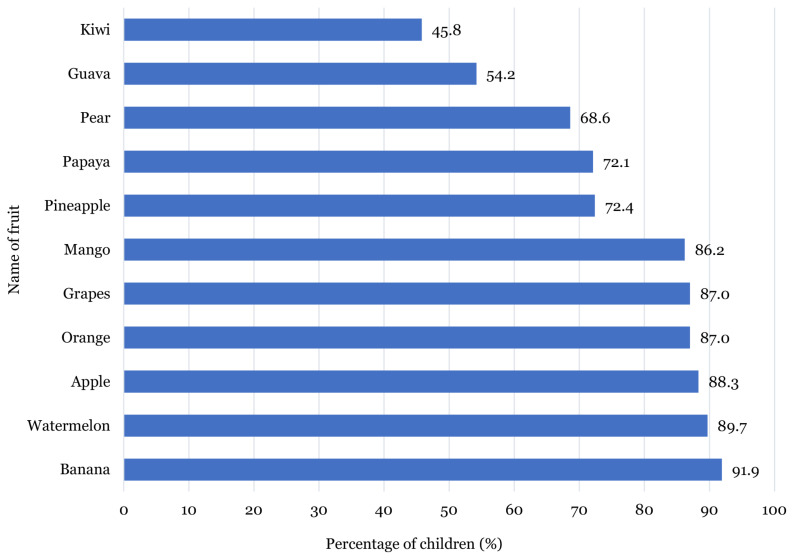
Percentage for fruit preferences among children

**Figure 2:**
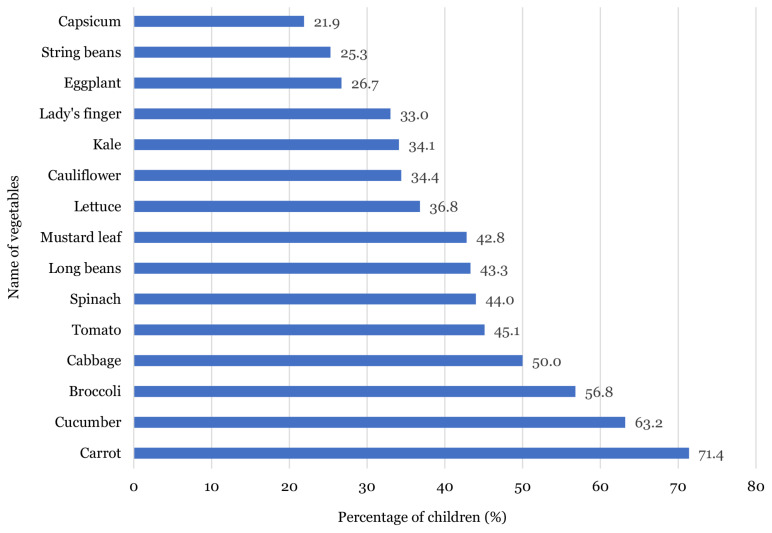
Percentage for vegetables preferences among children

Figure 1 shows that majority of the children preferred to eat bananas (91.9%) and watermelons (89.7%). More than 80.0% of the subjects liked to eat apples (88.3%), oranges (87.0%), grapes (87.0%) and mangoes (86.2%). Only 54.2% and 45.8% of the children liked to eat guavas and kiwis, respectively. As shown in Figure 2, carrots (71.4%), cucumbers (63.2%) and broccolis (56.8%) were the most preferred vegetables by the children, while string beans (25.3%) and capsicums (21.9%) were the least preferred.

### Attitudes on Fruits and Vegetables Intake

Based on Table 2, the children had a moderate attitude towards fruits and vegetables intake with a mean (SD) score of 70.3 (19.9). A majority (95.5%) of them agreed that they liked to eat fruits, whereas 70.1% agreed that they liked to eat vegetables. Moreover, 75.4% of the children preferred to try new fruits and ate a lot of fruits, while only 68.7% preferred to try new vegetables and ate lot of vegetables (66.4%). Ninety-one percent of the children believed that the daily consumption of fruits and vegetables keeps them healthy. However, almost half of them disagreed that they could not obtain complete nutrition by eating only one type of fruits (53.0%) or vegetables (56.7%).

### Recognition of Fruits and Vegetables

Based on Table 3, the overall mean (SD) score for the recognition of fruits and vegetables among children was at a moderate level of 76.8 (18.1). The most recognisable fruits were oranges (100.0%) and apples (99.3%), while the least identified fruits were guavas (68.7%) and pears (53.0%). Tomatoes (96.3%) and carrots (94.8%) were the most recognised vegetables, while kales (36.6%) and string beans (32.1%) were the least recognised. The pears were the least recognised fruits and was confused with guavas and rose apples. Conversely, string beans were the least recognised vegetables and were assumed as peas or short beans. The least recognised fruits that received the highest rating for not being eaten were kiwis (36.6%), guavas (36.6%) and pears (21.6%). The least recognised vegetables that received a high rating for not being eaten were capsicums (55.2%) and string beans (56.7%).

### Knowledge of Fruits and Vegetable Intake

As shown in Table 2, the overall nutrition knowledge of the fruits and vegetables intake among the children was moderate with a mean (SD) score of 73.6 (17.5). More than half of the children (53.0%) were not aware of the recommended intake of three servings of vegetables and two servings of fruits per day. In addition, more than 50.0% of the children were aware of the health benefits of fruits and vegetables consumption in preventing constipation (71.6%), strengthening teeth and bone (78.4%) and maintaining healthy skin (67.2%).

## Discussion

This study explores the preference, attitude, recognition and knowledge of fruits and vegetables consumption among Malay school-going children. A link between the least recognised fruits and vegetables and the least preferred fruits and vegetables among the children was identified in this study, whereby most of the children were not familiar with kiwi, guava and pear, thus resulting in low percentage of children liking these fruits. Similar findings were observed in the vegetable group, where kale, string bean and capsicum were the least preferred vegetables as well as the least recognised vegetables among the children. One possible reason that may account for this observation is the children’s familiarity and repeated exposure of the fruit and vegetables consumed (15, 18, 27). Therefore, familiarising children with fruits and vegetables through repeated exposure may improve their fruits and vegetables preferences and consumption.

Moreover, geographical differences could also influence the recognition of fruits and vegetables intake among Malay children (12). Malaysia has undergone a rapid nutrition transition characterised by a shift from a traditional grain-based diet that is high in complex carbohydrates and fibre to a diet high in animal-based produce, oils and fats, sweeteners and low in fibre (28–29). This transition is thought to affect the dietary pattern of the children and reduce the exposure to certain types of food such as fruits and vegetables. In a previous study by Norlida Mat et al. (24), the authors observed that the mean daily intake of dietary fibre in Malaysian adolescents who lived in rural areas was significantly higher as compared to urban adolescents. A possible reason for this observation is due to the high availability of plant sources in rural areas and increased availability of fast food in urban areas. Therefore, geographical differences may play a role in the recognition of fruits and vegetables among the children.

The taste of food was one of the strongest factors influencing the children’s preferences for fruits and vegetables. This study demonstrated that sweet fruits and vegetables such as banana, watermelon, apple, carrot and cucumber were most preferred by the children. In addition, the children in this study demonstrated a higher preference for fruits as compared to vegetables. This finding is supported by Dresler et al. (30), which reported that children preferred mild and sweet vegetables such as carrots, corns, potatoes, broccolis and cauliflowers. This factor may also influence the children’s preferences in consuming more fruits compared to vegetables owing to the bitter taste of vegetables (17). Moreover, food texture was also found to influence children’s preference for certain types of fruits and vegetables (31). It was suggested that children prefer soft and crunchy foods (31–32). Hence, this finding lends support to the observation in this study that shows the children’s preferences for eating certain fruits or vegetables such as apple, banana, carrot and cucumber.

The children in this study demonstrated a moderate score of 70.3% in their attitude towards the fruits and vegetables intake. Majority of the children agreed that they liked to eat fruits, while a lower percentage of children agreed that they liked to eat vegetables. Similarly, majority of the children’s attitudes based on their willingness to try new vegetables and consume more vegetables were lower as compared to the fruits intake. This finding is consistent with another study by Webb (33) who observed that a lower percentage of children strongly agreed that they liked eating vegetables as compared to fruits. This observation may be due to the bright colours and sweet taste of fruits, thus appearing more attractive as compared to vegetables. A study by Noradilah and Zahara (18) showed that a minimum of three exposures to vegetables increased the liking and consumption among the children. In addition, more than half of the children surveyed in this study expressed a reluctant attitude of eating a variety of fruits and vegetables since a high percentage of the children disagreed that they were not able to obtain complete nutrition by eating only one type of fruit and vegetable. It was previously shown that the consumption of a variety of fruits and vegetables was associated with higher diet quality among pre-schoolers (34). Therefore, it is important to educate and encourage children regarding the importance of eating a variety of fruits and vegetables.

The results of this study showed that Malay children had a moderate (73.6%) knowledge level regarding fruit and vegetable intake. This level was higher as compared to the study by Bundhun et al. (35) who reported a nutrition knowledge level of 53.3% regarding the fruit and vegetable intake among the Mauritian childhood population. This difference could be attributed to the study population of Malay children from urban areas as compared to the study population investigated by Bundhun et al. (35) that was composed of children from rural areas in Mauritius. A study by Naeeni et al. (36) also showed that children and adolescents in urban Isfahan had a higher nutrition knowledge level compared to those in rural areas. Therefore, geographical factors are likely to affect the children’s nutrition knowledge of fruits and vegetables consumption. In addition, majority of the children surveyed in this study were knowledgeable of the health benefits of fruits and vegetables. Based on the Health Belief Model (37), perceived benefits are one of the key components that influence an individual to take preventive action. Nevertheless, despite the understanding of the health benefits of fruits and vegetables demonstrated by the children in this study, certain factors possibly contributed to the low consumption of fruits and vegetables among Malaysian children such as the low availability of fruits and vegetables at home (29) and low socioeconomic status (38). In Malaysia, children aged between 4 and 6 years with a low socioeconomic status had a lower mean intake of fruits as compared to the high-income groups, thus resulting in the limited accessibility of high quality and healthy diet (38). Therefore, intervention programmes involving parent participation should be implemented and parents should strive to create a supportive home environment by ensuring the availability of fruits and vegetables at home to increase the children’s consumption of fruits and vegetables.

Additionally, this study revealed that the children lacked specific knowledge regarding the recommended serving sizes of fruits and vegetables. A similar finding was reported by Norlida Mat et al. (24), whereby majority of Malaysian adolescents had limited knowledge of the recommended serving sizes of fruits and vegetables to be consumed daily. This finding may also contribute to the low consumption of fruits and vegetables among Malaysian children since they are not aware of portion sizes of fruits and vegetables that should be consumed daily. Therefore, nutrition education programmes should aim to educate children on the recommended serving sizes of fruits and vegetables. Likewise, intervention programmes focusing on play-based learning methods such as the use of educational board games are also effective in improving children’s nutrition knowledge and dietary behaviours (39).

Nevertheless, several limitations have been identified in this study. Firstly, only a descriptive analysis of the data has been obtained from this study and thus, the cause and effect of the findings have not been determined in this study. Furthermore, this study was only carried out among the Malay children residing in Kuala Lumpur and therefore, may not be representative of the actual children population in Malaysia. Moreover, the selection of children based on the teacher’s decision may lead to a selection bias that reduces the validity of the findings. Finally, the actual fruits and vegetables intake among the children was not measured in this study as children may not be able to perform an accurate diet recall. Therefore, the association of preference, attitude, recognition and knowledge regarding the consumption of fruits and vegetables was not performed. Despite these limitations, the outcomes of this study highlight the existing gap that may be utilised by health professionals to develop an effective intervention programme to increase consumption of fruits and vegetables among children.

## Conclusion

The preference and recognition of fruits were higher as compared to vegetables among the Malay children surveyed in this study. The overall attitude, recognition and knowledge regarding the fruits and vegetables intake among Malay children were at a moderate level. However, the children were not aware of the recommended serving size of fruits and vegetables and the consumption of a variety of fruits and vegetables. There is a need for further research to be performed on the factors that contribute to the low preference and recognition towards vegetables as well as the moderate level attitude, recognition and knowledge regarding fruits and vegetables intake among Malay children. In addition, the recruitment of subjects for future research should cover a wider geographical area and diverse ethnic groups to elucidate the actual scenario of preference, attitude, recognition and knowledge of the fruits and vegetables intake among the children in Malaysia. The outcomes of this study indicate that intervention programmes should be aimed at improving children’s nutrition knowledge on the serving size and consumption of a variety of fruits and vegetables.

## Figures and Tables

**Table 1:** Socio-demographic characteristics of children

Characteristics	Mean (SD)	*n* (%)
Age (years)	10.3 (1.0)	
Gender		
Male		70 (52.2)
Female		64 (47.8)
Father’s education level		
Primary school		23 (17.2)
Secondary school		83 (61.9)
Tertiary school		28 (20.9)
Mother’s education level		
Primary school		14 (10.4)
Secondary school		75 (56.0)
Tertiary school		45 (33.6)
Monthly household income		
< RM3,900		95 (70.9)
≥ RM3,900		39 (29.1)

**Table 2:** Attitude and knowledge on fruit and vegetable intake among children

Questions	Answer *n* (%)		Total score mean (SD)

	Yes	No	
Attitude			
I like to eat vegetables	94 (70.1)	40 (29.9)	70.3 (19.9)
I like trying new vegetables	92 (68.7)	42 (31.3)	
I try to eat lot of vegetables	89 (66.4)	45 (33.6)	
Eating vegetables every day keeps me healthy	122 (91.0)	12 (9.0)	
I like to eat fruit	128 (95.5)	6 (4.5)	
I like trying new fruit	101 (75.4)	33 (24.6)	
I try to eat lot of fruit	101 (75.4)	33 (24.6)	
Eating fruit every day keeps me healthy	122 (91.0)	12 (9.0)	
Eating fruit and vegetables will prevent constipation	86 (64.2)	48 (35.8)	
I cannot get a complete nutrient by eating only one types of vegetable	58 (43.3)	76 (56.7)	
I cannot get a complete nutrient by eating only one types of fruit	63 (47.0)	71 (53.0)	

Knowledge			
Fruit and vegetables are high fibre foods	81 (60.4)	53 (39.6)	73.6 (17.5)
Fruit and vegetables can prevent constipation	96 (71.6)	38 (28.4)	
Fruit and vegetables can strengthen teeth and bone	105 (78.4)	29 (21.6)	
Fruit and vegetables are rich with vitamins	123 (91.8)	11 (8.20)	
Papaya is a type of fruit	130 (97.0)	4 (3.0)	
Spinach is a type of vegetable	123 (91.8)	11 (8.20)	
Orange are rich with Vitamin C	104 (77.6)	30 (22.4)	
Fruit and vegetables can make my skin look healthy	90 (67.2)	44 (32.8)	
Everyone has to eat three servings of vegetables every day	63 (47.0)	71 (53.0)	
Everyone has to eat two servings of fruit every day	62 (46.3)	72 (53.7)	
Everyone has to eat various types of fruit and vegetables every day	110 (82.1)	24 (17.9)	

**Table 3:** Recognition of fruits and vegetables

Fruits and vegetables	Correct	Incorrect	Never tried	Alternative names	Total score mean (SD)

	*n* (%)	*n* (%)	*n* (%)		
Fruits					
Orange	134 (100.0)		8 (6.0)		
Apple	133 (99.3)	1 (0.7)	4 (3.0)		
Banana	131 (97.8)	4 (2.2)	7 (5.2)		
Mango	129 (96.3)	5 (3.7)	10 (7.5)		
Watermelon	128 (95.5)	6 (4.5)	7 (5.2)		
Grape	127 (94.8)	7 (5.2)	14 (3.0)		
Pineapple	127 (94.8)	7 (5.2)	20 (15.0)		
Papaya	126 (94.0)	8 (6.0)	15 (11.2)		
Kiwi	99 (73.9)	35 (26.1)	49 (36.6)	Lemon, avocado, *ciku*	
Guava	92 (68.7)	42 (31.3)	49 (36.6)	Pickles	
Pear	71 (53.0)	63 (47.0)	29 (21.6)	Guava, rose apple	
Vegetables					
Tomato	129 (96.3)	5 (3.7)	36 (26.9)		
Carrot	127 (94.8)	7 (5.2)	18 (13.4)		76.8 (18.1)
Cucumber	122 (91.0)	12 (9.0)	31 (23.1)		
Broccoli	112 (83.6)	22 (16.4)	23 (17.2)		
Long beans	111 (82.8)	23 (17.2)	40 (29.9)		
Eggplant	111 (82.8)	23 (17.2)	60 (44.8)		
Cabbage	110 (82.1)	24 (17.9)	38 (28.3)		
Lady’s finger	100 (74.6)	34 (25.4)	52 (38.8)		
Spinach	76 (56.7)	58 (43.3)	41 (30.6)	Mustard, kale	
Mustard leaf	75 (56.0)	59 (44.0)	46 (34.3)	Spinach	
Lettuce	66 (49.3)	68 (50.7)	54 (40.3)	Vegetable eaten with chicken rice	
Cauliflower	63 (47.0)	71 (53.0)	59 (44.0)	Broccoli	
Capsicum	54 (40.3)	80 (59.7)	74 (55.2)	Chilli	
Kale	49 (36.6)	85 (63.4)	30 (22.4)	Cassava leaves, *ulam*	
String beans	43 (32.1)	91 (67.9)	76 (56.7)	Peas, short bean	
